# High baseline fat mass, but not lean tissue mass, is associated with high intensity low back pain and disability in community-based adults

**DOI:** 10.1186/s13075-019-1953-4

**Published:** 2019-07-05

**Authors:** Sharmayne R. E. Brady, Donna M. Urquhart, Sultana Monira Hussain, Andrew Teichtahl, Yuanyuan Wang, Anita E. Wluka, Flavia Cicuttini

**Affiliations:** Department of Epidemiology and Preventive Medicine, School of Public Health and Preventive Medicine, Monash University, 553 St Kilda Rd, Melbourne, Victoria 3004 Australia

**Keywords:** Low back pain, Body composition, Fat mass, Lean tissue mass, Android

## Abstract

**Objectives:**

Low back pain is the largest contributor to disability worldwide. The role of body composition as a risk factor for back pain remains unclear. Our aim was to examine the relationship between fat mass and fat distribution on back pain intensity and disability using validated tools over 3 years.

**Methods:**

Participants (aged 25–60 years) were assessed at baseline using dual-energy X-ray absorptiometry (DXA) to measure body composition. All participants completed the Chronic Pain Grade Scale at baseline and 3-year follow-up. Of the 150 participants, 123 (82%) completed the follow-up.

**Results:**

Higher baseline body mass index (BMI) and fat mass (total, trunk, upper limb, lower limb, android, and gynoid) were all associated with high intensity back pain at either baseline and/or follow-up (total fat mass: multivariable OR 1.05, 95% CI 1.01–1.09, *p* < 0.001). There were similar findings for all fat mass measures and high levels of back disability. A higher android to gynoid ratio was associated with high intensity back pain (multivariable OR 1.04, 95% CI 1.01–1.08, *p* = 0.009). There were no associations between lean mass and back pain.

**Conclusions:**

This cohort study provides evidence for the important role of fat mass, specifically android fat relative to gynoid fat, on back pain and disability.

## Key messages

1. This longitudinal study examined the role of fat mass and distribution, using DXA, on back pain outcomes.
2. The results of this study suggest targeting specifically a reduction in fat mass (not just simply weight loss) to prevent back pain, particularly in those who carry excess fat in the android distribution.

## Background

Low back pain is of major public health significance worldwide, with the recent global burden of disease study indicating it is now the leading cause of global disability, ahead of 290 other conditions [1]. Not only is low back pain highly disabling and prevalent [2], but it is associated with considerable financial burden [3]. Studies show that high levels of low back pain intensity and persistent back pain are strong independent predictors of higher financial burden [4]. Hence, it is important to identify modifiable risk factors for persistent, high intensity low back pain, which in turn will inform preventive strategies.

Obesity, a global epidemic, is associated with a myriad of complications, many of which are musculoskeletal in nature [5]. The relationship between obesity and back pain has previously been reviewed [6, 7], and while obesity was found to be weakly associated with back pain, these studies only used weight, body mass index (BMI), waist circumference, and waist-hip ratio as measures of obesity. Such measures do not differentiate accurately between fat mass and lean tissue mass (or muscle mass), nor do they estimate fat mass at different sites around the body.

Previous studies demonstrate that fat mass and muscle mass have quite different roles in the pathogenesis of musculoskeletal disease [8–10]. In cross-sectional studies of community-based cohorts, those with greater fat mass had higher levels of back pain intensity and disability, whereas greater lean tissue mass was not associated with back pain intensity or disability [10, 11]. Similarly, increased fat mass was associated with a higher risk of Modic type 2 changes in the spine which are associated with low back pain, whereas fat-free mass tended to be protective [9]. Hence, it is important to accurately differentiate fat mass from lean tissue (or muscle) mass when evaluating their impact on back pain.

There are many options available for measuring body composition in research and clinical settings; however, dual-energy X-ray absorptiometry (DXA) is considered the gold standard method for analyzing body composition [12, 13], with excellent precision, reproducibility, and reliability between operators [12, 14]. Although its use is limited by cost and accessibility, requiring a certified radiology technician [15, 16], DXA can provide a rapid, non-invasive estimation of body composition with minimal radiation. Another method of estimating body composition is by using bioelectrical impedance analysis, which is fast and inexpensive and does not require extensive operator training; however, it relies on body hydration, which is difficult to assess and may differ between participants [17]. Subcutaneous fat thickness measurement devices such as skinfold calipers are non-invasive, inexpensive, and practical. However, accurate measurement can be challenging, especially in large individuals [18]. Also, tissue compressibility differs between men and women, and there can be significant inter- and intra-observer variability among inexperienced operators [19], thus limiting its use. Hence, while there are several options available to estimate body composition, DXA is the most valid and reproducible.

A recent systematic review reported that there were insufficient cohort studies available to perform a meta-analysis and draw conclusions regarding the relationship between fat mass and risk of incident and worsening pain, highlighting the need for further high-quality longitudinal studies [20]. Of the prospective studies that have assessed body composition in relation to back pain, only one used DXA [21], two studies used bioelectrical impedance analysis [17, 22], and one used a subcutaneous fat thickness-measuring device [23]. Moreover, the results from these longitudinal studies have been inconsistent, with only two studies showing a positive association between fat mass or body fat percentage and back pain after multivariable analysis [17, 23]. Also, it is well recognized that fat located at different sites, such as with gynoid or android distribution, have different metabolic effects [24]. No previous longitudinal study has examined whether fat distribution, assessed using sensitive methods such as DXA, affects back pain differently. This study aimed to explore the association between fat mass and fat distribution on back pain intensity and disability over a 3-year period, using well-validated tools to measure both back pain (chronic pain grade questionnaire) and body composition (DXA).

## Methods

### Study population

Participants were recruited to partake in a study examining musculoskeletal health [25] and were not selected based on their pain status or for seeking treatment for their pain. Recruitment was through local media and public, private, and community weight loss clinics. Subjects were excluded if they had a history of any arthropathy diagnosed by a medical practitioner, prior surgical intervention to the knee including arthroscopy, and previous significant knee injury requiring non-weight bearing therapy or requiring prescribed analgesia, malignancy, or contraindication to magnetic resonance imaging. Participants (*n* = 150) who underwent DXA within 12 months of answering the Chronic Pain Grade Scale in 2008–2009 were eligible for the current study. Of these, 123 (82%) completed the same back pain questionnaire approximately 3 years later in 2011–2012.

### Anthropometric measures

Measures of obesity taken at the time that baseline back pain was assessed were used in the current analysis. Body composition was analyzed by two experienced radiographers using DXA and operating system version 9 (Monash Medical Centre, Clayton Victoria, Australia, and Austin Hospital, Heidelberg, Victoria, Australia, GE Lunar Prodigy, GE Lunar Corp., Madison, WI). The machine has a weight limit of approximately 160 kg. Weight was measured to the nearest 0.1 kg using DXA (shoes, socks, and bulky clothing were removed). Height at baseline was measured to the nearest 0.1 cm (shoes and socks were removed) using a stadiometer. From these data, BMI was calculated and reported in kilograms per meter square. Android fat mass relates to the distribution of excess fat around the abdomen, whereas gynoid fat mass relates to the distribution of excess fat around the hips, thighs, and buttocks. The android and gynoid ratio (%) was calculated using the formula: android fat mass (kg)/gynoid fat mass (kg) × 100.

### Mental health

The Short Form 36 (SF-36) is a commonly used and well-validated tool to assess health-related quality of life [26]. The mental component summary (MCS) of the SF-36 was used to examine psychological health and well-being in participants in the baseline survey. High MCS scores indicate an absence of psychological distress and a lack of limitations in social activities due to emotional problems [26].

### Physical activity

Participants were asked “On how many days during the last 14 days did you spend at least 20 minutes doing strenuous exercise such as bicycling, brisk walking, that was severe enough to raise your pulse rate or cause you to breathe faster?,” with frequency options as follows: “no days, 1 to 2 days, 3 to 5 days, 6 to 8 days, or 9 or more days.” In this study, strenuous physical activity was defined as undertaking strenuous physical activity for at least 20 min, on at least 1 or more days over the previous 14 days [10, 25].

### Low back pain

The Chronic Pain Grade Scale was used to quantify low back pain intensity and disability [27]. It is a seven-item, self-administered questionnaire. High intensity back pain was defined as pain intensity score ≥ 50 out of 100, and high disability back pain was defined as disability points of three or more, out of a maximum of six [27]. “Resolving” high intensity back pain was defined as reporting high intensity back pain in the baseline survey but not in the follow-up survey, and “developing” high intensity back pain was defined as reporting high intensity back pain in the follow-up survey but not in the baseline survey. “Persistent” high intensity back pain was defined as reporting high intensity back pain in both baseline and follow-up surveys, whereas “no” high intensity back pain was defined as reporting no high intensity back pain in either survey. The four high disability back pain groups were defined using the same approach as for the four high intensity back pain groups.

### Statistical analysis

Comparisons of mean values for continuous variables across the four groups (no, developing, resolving, and persistent back pain) were obtained using ANOVA. Differences in categorical variables across the four back pain groups were obtained using chi-squared test. As there were no significant differences in baseline body composition measures among those with resolving, developing, or persistent high intensity back pain (all *p* > 0.05), we combined these three groups for analysis (i.e., any high intensity back pain vs. no high intensity back pain over the study period). Similarly, the anthropometric measures for those with any high disability back pain at either baseline or follow-up were also very similar (data not shown); hence, they were also grouped together into any or no high disability back pain over the study period in Table 3. Binary logistic regression (odds ratios with 95% confidence intervals) was used to analyze the relationship between body composition and any high intensity/disability back pain over the study period, after adjustment for confounders (age, sex, strenuous physical activity, MCS of SF-36, and fat or lean tissue mass measure). When performing multivariable analyses for fat mass (total, trunk, android, and gynoid), total lean tissue mass was included as a potential confounder. When performing multivariable analyses for upper and lower limb fat mass, lean tissue mass in the upper and lower limb respectively was included as a potential confounder. *p* values less than 0.05 (two-tailed) were regarded as statistically significant. All analyses were performed using the statistical package SPSS (standard version 24.0) or STATA SE 13.0.

## Results

Due to the weight limit of the DXA machine, one participant was excluded from the study. Of the 150 participants who completed the baseline back pain questionnaire and DXA, 123 (82%) completed the follow-up back pain questionnaire approximately 3 years later. There were no major differences in baseline age (mean [SD] 48.6 [8.5] vs. 47.9 [9.0] years), gender (percentage female 78.1% vs. 82.0%), and BMI (31.9 [8.3] vs. 32.7 [8.6] kg/m^2^) between those who completed and did not complete the follow-up back pain questionnaire.

Of the 123 participants, mean (SD) age was 48.6 (8.5) years, they were primarily female (78.0%), and mean BMI was in the obese category (32.0 kg/m^2^). Twenty-eight (22.8%) and 26 (21.1%) participants experienced high intensity back pain at baseline and follow-up, respectively. The baseline characteristics are presented in Table 1, according to whether they had no, resolving, developing, or persistent high intensity back pain over the 3-year study period. A total of 39 (31.7%) participants reported high intensity back pain at either the baseline or follow-up survey or both, with 15 (12.2%) experiencing persistent levels of high intensity back pain, 11 (8.9%) developing high intensity back pain, and 13 (10.6%) having resolving back pain over the study period. Those who had experienced high intensity back pain at any time point had a higher mean BMI, compared to those who had not experienced any high intensity back pain [mean BMI (SD) for resolving pain 36.5 (7.5), developing pain 37.0 (9.5), persistent pain 36.4 (7.9), versus no high intensity back pain 29.7 (7.5)] (*p* < 0.001). Those experiencing high intensity back pain at any time point also had poorer mental health status than those without any high intensity back pain during the study [mean MCS (SD) for resolving pain 42.1 (15.6), developing pain 42.0 (16.9), persisting pain 40.5 (15.0), and no high intensity back pain 48.9 (11.7)] (*p* = 0.003).

**Table 1:** Characteristics of participants with no, resolving, developing, and persistent high intensity back pain

Baseline characteristics	High intensity low back pain			
	No (*n* = 84)	Resolving (*n* = 13)	Developing (*n* = 11)	Persistent (*n* = 15)
Age (years)	49.0 (8.2)	42.6 (10.6)	50.9 (6.6)	50.1 (7.7)
Gender (*n*, % female)	64 (76.2)	10 (76.9)	10 (90.9)	12 (80.0)
BMI (kg/m^2^)	29.7 (7.5)	36.5 (7.5)	37.0 (9.5)	36.4 (7.9)
SF-36 mental component summary score	48.9 (11.7)	42.1 (15.6)	42.0 (16.9)	40.5 (15.0)
Strenuous physical activity for > 20 min (over 14-day period) (*n*, %)	70 (83.3)	9 (69.2)	9 (81.8)	7 (46.7)
Fat mass (kg)				
Total	31.6 (16.1)	46.4 (16.4)	45.7 (14.6)	43.1 (14.6)
Trunk	16.5 (8.4)	25.1 (8.2)	24.1 (7.2)	23.0 (8.1)
Upper limb	3.0 (1.7)	4.3 (2.1)	4.5 (1.9)	4.3 (1.6)
Lower limb	11.2 (6.4)	15.9 (7.0)	16.1 (5.8)	14.8 (6.0)
Android	2.9 (1.6)	4.5 (1.7)	4.2 (1.5)	4.2 (1.8)
Gynoid	5.8 (2.6)	8.0 (2.9)	8.0 (2.3)	7.7 (2.4)
Android to gynoid ratio (%)	48.6 (16.2)	58.3 (14.2)	52.5 (8.4)	54.2 (17.8)
Lean tissue mass (kg)				
Total	46.8 (9.5)	51.9 (11.7)	48.1 (10.2)	49.0 (12.2)
Upper limb	5.1 (1.4)	5.7 (1.4)	5.0 (1.3)	5.5 (1.9)
Lower limb	15.4 (3.4)	17.1 (3.4)	15.6 (3.1)	15.7 (4.5)
Total body fat to lean mass ratio	0.7 (0.3)	0.9 (0.3)	0.9 (0.2)	0.9 (0.3)

Mean (SD) unless otherwise noted

*BMI* body mass index, *SF-36* Short Form 36

In relation to body composition measures, those who had experienced any high intensity back pain during the study had higher mean total, truncal, upper limb, lower limb, android, and gynoid fat mass at baseline, than those without any high intensity back pain during the study [mean total body fat mass (SD) 46.4 (16.4) for resolving pain, 45.7 (14.6) for developing pain, 43.1 (14.6) for persisting pain, and 31.6 (16.1) for no high intensity back pain] (all *p* < 0.001). Similarly, those with any high intensity back pain had a higher fat to lean mass ratio than those without any high intensity back pain [mean (SD) total fat to lean mass ratio 0.9 (0.3) for resolving pain, 0.9 (0.2) for developing pain, 0.9 (0.3) for persisting pain, and 0.7 (0.3) for no high intensity back pain] (*p* = 0.0001). There was no association between baseline lean tissue mass across the high intensity back pain groups. As there were no significant differences in any body composition measure between those with persistent, resolving, or developing high intensity back pain, we combined these three groups together for analysis purposes (all *p* > 0.05). Those with high intensity back pain at either time point were also more likely to experience high disability back pain than those without any high intensity back pain (data not shown).

The relationships between body composition and any high intensity back pain are presented in Table 2, after adjustment for confounding variables. With increasing BMI and fat mass (at all body regions), there was a higher risk of high intensity back pain over the study period. For example, for every 1-kg increase in total body fat mass at baseline, there was a 5% increased risk of high intensity back pain over the study period (OR 1.05, 95% CI 1.01–1.09, *p* = 0.01) after adjusting for age, gender, strenuous physical activity, total lean tissue mass, and mental health. Similar relationships were evident for trunk, upper limb, and lower limb fat mass, as well as for the android and gynoid distribution of fat. The association between android fat mass and high intensity back pain appeared stronger than the association for gynoid fat mass (android fat mass OR 1.60, 95% CI 1.13–2.26; gynoid fat mass OR 1.30, 95% CI 1.04–1.61), but this was not statistically significant. A higher android to gynoid ratio was significantly associated with an increased risk of high intensity back pain, which persisted after adjustment for multiple confounders. No significant relationship was evident for lean tissue mass.

**Table 2:** The relationship between body composition and any high intensity low back pain during the study, after adjustment for confounders

	Multivariable odds ratio^a^	95% CI	*p* value
BMI^b^	1.10	1.05–1.17	< 0.001
Fat mass			
Total	1.05	1.01–1.09	0.01
Trunk	1.11	1.04–1.20	0.003
Upper limb	1.44	1.07–1.93	0.02
Lower limb	1.09	1.00–1.19	0.05
Android	1.60	1.13–2.26	0.008
Gynoid	1.30	1.04–1.61	0.02
Android to gynoid ratio (%)^c^	1.04	1.01–1.08	0.009
Lean tissue mass			
Total	1.00	0.93–1.08	0.92
Upper limb	1.59	0.92–2.74	0.10
Lower limb	1.05	0.85–1.28	0.67
Fat to lean mass ratios^c^			
Total body fat to lean mass ratio (per 10 kg)	1.33	1.13–1.58	0.001
Upper limb fat to lean mass ratio (per 10 kg)	1.21	1.05–1.40	0.008
Lower limb fat to lean mass ratio (per 10 kg)	1.20	1.05–1.37	0.007

^a^Relationship between body composition and any high intensity back pain (at any time point), adjusted for age, gender, strenuous physical activity, and fat or lean tissue mass measure, in addition to mental health component score (from SF-36). When performing multivariable analyses for fat mass (total, trunk, android, and gynoid), total lean tissue mass was included as a potential confounder. When performing multivariable analyses for upper and lower limb fat mass, lean tissue mass in the upper and lower limb respectively was included as a potential confounder

^b^BMI adjusted for age, gender, strenuous physical activity, and mental health component score (from SF-36)

^c^All ratios were adjusted for age, gender, strenuous physical activity, and mental health component score (from SF-36)

Table 3 presents the relationship between body composition and high disability back pain at either baseline and/or follow-up. With increasing BMI and fat mass (at all body regions), there was an increased risk of high disability back pain over the study period. For example, for every 1-kg increase in total body fat mass, there was a 6% increased risk of high disability back pain (multivariable OR 1.06, 95% CI 1.01–1.011, *p* = 0.02), after adjusting for potential confounders. Similar relationships were evident for fat mass in all other body regions. Although not significant, having an android distribution of fat tended to be associated with higher risk of high disability back pain (OR 1.65, 95% CI 1.07–2.56) than those with a gynoid distribution (OR 1.40, 95% CI 1.07–1.84). A higher android to gynoid ratio was associated with a greater risk of high disability back pain, but after adjustment for multiple confounders, the association was no longer statistically significant (*p* = 0.11). Increased fat to lean mass ratios across all regions were also associated with significantly higher risk of high disability back pain at either survey. However, no significant relationship was evident for lean tissue mass.

**Table 3:** The relationship between body composition and any high disability back pain during the study, after adjustment for confounders

	Multivariable odds ratio^a^	95% CI	*p* value
BMI^b^	1.12	1.04–1.20	0.002
Fat mass			
Total	1.06	1.01–1.11	0.02
Trunk	1.11	1.01–1.21	0.02
Upper limb	1.53	1.07–2.19	0.02
Lower limb	1.12	1.00–1.25	0.05
Android	1.65	1.07–2.56	0.03
Gynoid	1.40	1.07–1.84	0.02
Android to gynoid ratio (%)^c^	1.03	0.99–1.07	0.11
Lean tissue mass			
Total	0.98	0.89–1.08	0.73
Upper limb	1.13	0.55–2.30	0.74
Lower limb	1.01	0.78–1.32	0.93
Fat to lean mass ratios^c^			
Total body fat to lean mass ratio (per 10 kg)	1.33	1.08–1.64	0.007
Upper limb fat to lean mass ratio (per 10 kg)	1.28	1.06–1.54	0.009
Lower limb fat to lean mass ratio (per 10 kg)	1.23	1.04–1.45	0.01

^a^Relationship between body composition and any high disability back pain (at any time point), adjusted for age, gender, strenuous physical activity, and respective fat or lean tissue mass measure in addition to mental health component score (from SF-36). When performing multivariable analyses for fat mass (total, trunk, android, and gynoid), total lean tissue mass was included as a potential confounder. When performing multivariable analyses for upper and lower limb fat mass, lean tissue mass in the upper and lower limb respectively was included as a potential confounder

^b^BMI adjusted for age, gender, strenuous physical activity, and mental health component score (from SF-36)

^c^All ratios were adjusted for age, gender, strenuous physical activity, and mental health component score (from SF-36)

## Discussion

In this cohort study of community-based adults aged 25–60 years, all measures of baseline fat mass (including total, upper limb, lower limb, trunk, android, gynoid) were associated with a higher risk of having any high intensity back pain over the 3-year study period. A similar relationship was present for high disability back pain. These relationships remained significant after adjusting for potential confounders, including age, gender, lean tissue mass, mental health status, and physical activity. A higher android to gynoid ratio was also significantly associated with high intensity back pain. This longitudinal study, using DXA to assess body composition, not only supports the role of body fat mass in the pathogenesis of back pain and disability, but also suggests that android fat distribution relative to gynoid has a role in high intensity back pain.

In the current study, we found that baseline fat mass, across all body regions, was associated with both any high intensity back pain (present at either baseline and/or 3-year follow-up) and any high levels of low back disability over the study period. Many previous studies demonstrating a relationship between obesity and back pain have been cross-sectional [6, 7, 10, 11]. Of the prospective studies, many of which are occupational cohort studies, most have only measured body mass index as the measure of obesity [28–39]. Four prospective studies that have measured body composition using methods such as DXA [21], bioelectrical impedance analysis [17, 22], and subcutaneous fat thickness-measuring devices [23] have demonstrated inconsistent results. Hussain et al. showed in a population-based, longitudinal study of 4986 Australian adults that fat mass and percent fat mass, measured using bioelectrical impedance analysis, were positively associated with back pain intensity and disability, independent of fat-free mass [17]. In contrast, in the other longitudinal study of 314 Spanish adult twins, there was no increased risk of back pain with increasing fat mass percentage [22]. The longitudinal, population-based study of 1099 older adults, which utilized DXA to more accurately measure fat mass, found no significant association between increased fat mass, fat mass index, and back pain in multivariable analysis, but did find significant associations for multi-site pain [21]. However, they did not use a well-validated back pain questionnaire, nor did they explore the effects of fat at different sites around the body [21]. Using DXA to explore the effects of fat at several different body regions and a well-validated back pain measure of intensity and disability, we have provided strong evidence for the association of fat mass, but not lean mass on back pain, supporting the hypothesis of a systemic etiology of back pain.

The current study is the first to demonstrate the importance of the location of adipose tissue in relation to back pain longitudinally. In this study, the android to gynoid ratio was significantly associated with high intensity back pain, suggesting that the android distribution of fat may be particularly important in the pathogenesis of back pain. It is well known that an android distribution of fat is associated with an increased risk of cardiovascular disease and metabolic disturbances [40, 41]. This is thought to be related to unique properties of abdominal adipocytes, such as site-specific differences in adipocyte size, physiology (e.g., catecholamine sensitivity, lipolysis, and insulin), and biochemistry (e.g., leptin, plasminogen activator inhibitor-1, and the renin-angiotensin system) [41, 42]. Android fat distribution is also associated with musculoskeletal conditions such as foot pain and disability [43] and reduced knee cartilage volume [44]. In the back pain literature, android distribution of fat has previously been associated with spinal structures, specifically Modic type 2 changes [9]. Modic type 2 changes, which are seen on MRI, histologically represent fatty replacement of the bone marrow [45] and are associated with reduced intervertebral disc height in the lumbar spine [9] and back pain [46]. Modic type 2 changes may represent a link between the android distribution of adiposity and back pain and warrant further examination.

The mechanism underpinning the relationship between increased fat mass, particularly in the android distribution, and back pain intensity and disability may occur via mechanical loading of body mass onto spinal structures. However, given the absence of an association between lean tissue mass and back pain, this suggests that a systemic or metabolic process is also at play, as opposed to a purely mechanical process. This is further supported by our findings of an association with android fat mass. Consistent with these findings, other studies have demonstrated an association between greater fat mass and pain in a range of musculoskeletal regions such as the hands [21], feet [43], and lower body pain sites [21, 47]. The finding that non-weight bearing joints are also associated with fat mass further supports a role for systemic or metabolic factors. It is now well recognized that adipose tissue is highly metabolically active, secreting both adipokines and inflammatory cytokines, creating a state of chronic low-grade inflammation [48]. The adipokine leptin has even been proposed as a possible causative link between obesity and osteoarthritis [49, 50], being associated with structural knee joint abnormalities [51], pain pathways [52], and an increased prevalence of back pain in women [53]. In addition, recently, the adipokine adipsin has been associated with back pain, independently of adiposity [54]. Together, this supports the notion that increased fat mass may be contributing to the pathogenesis of back pain, via metabolic pathways.

A limitation to the current study was that the study sample had fewer men in comparison to women. Despite our moderate sample size, we were able to demonstrate statistically significant relationships between all the fat mass measures and back pain intensity and disability. While we were not able to adjust for depression in our multivariable analysis, a known predictor for back pain, we were able to adjust for the mental component of the SF-36 score, which has been shown to identify 87% of patients with depression in a study of patients with chronic spinal pain [55]. Unfortunately, we were not able to report on back pain duration, treatments such as surgery, or the presence of compensation [56]. A further potential limitation was that we were unable to account for the effects of genetic factors and environmental influences [22], nor were we able to utilize a standardized questionnaire to measure physical activity level. Although we recruited from weight loss clinics, the proportion of overweight or obese individuals in our baseline sample (76%) is similar to that of the current Australian population (63%). The study has a number of strengths including our ability to control for multiple potential confounders, use of a sensitive tool for the measurement of body composition, and a well-validated questionnaire to quantify back pain intensity and disability.

## Conclusions

Given the huge burden of back pain globally [1, 3] and the lack of effective therapies available [57], identifying modifiable risk factors is crucial in order to target preventive strategies. Many previous studies that have explored the relationship between obesity and back pain have used body mass index [6, 7], which fails to distinguish fat mass from muscle mass. However, these two tissue types clearly play very different roles in the pathogenesis of musculoskeletal disease [9, 10]. This longitudinal study shows that all DXA measures of fat mass across several different body regions (total, upper limb, lower limb, truncal, android, and gynoid) were associated with a significantly higher risk of high intensity back pain and disability over 3 years, whereas there was no significant relationship for lean tissue mass. The results of this study suggest targeting specifically a reduction in fat mass (not just simply weight loss) to prevent back pain, particularly in those who carry excess fat in the android distribution. By targeting adiposity on a population level, it has the potential to lessen the burden of high intensity and disability back pain in overweight and obese adults.

## Data Availability

The datasets analyzed during the current study are not publicly available but are available from the corresponding author on reasonable request.

## Abbreviations

BMI: Body mass index
DXA: Dual-energy X-ray absorptiometry
MCS: Mental component summary
SF-36: Short Form 36
